# Accelerating RRT* convergence with novel nonuniform and uniform sampling approach

**DOI:** 10.1038/s41598-025-09992-y

**Published:** 2025-08-04

**Authors:** Sivasankar Ganesan, Mohanraj Thangamuthu, Balakrishnan Ramalingam, Madan Mohan Rayguru, Sethu Narayanan Tamilselvan

**Affiliations:** 1 Department of Mechanical Engineering, Amrita School of Engineering, Coimbatore, Amrita Vishwa Vidyapeetham, Coimbatore, India; 2 School of Electronics Engineering, Vellore Institute of Technology, Chennai, India; 3 Louisville Automation and Robotics Research Institute, University of Louisville, Louisville, KY USA; 4 Singapore University of Technology and Design, Singapore, Singapore

**Keywords:** Nonuniform–uniform sampler, RRT*, Path planning, Navigation, Autonomous mobile robot, Electrical and electronic engineering, Mechanical engineering

## Abstract

Path planning plays a crucial role in autonomous mobile robotics. Sampling-based path planners are widely and frequently employed to generate collision-free paths between a start and goal location. Due to its asymptotic optimality, the optimal rapidly-exploring random tree (RRT*) algorithm is the most widely used among these. However, its reliance on uniform sampling often results in slow convergence. To address this issue, this work proposes a novel hybrid sampling method called RRT*-NUS (nonuniform–uniform sampler), which combines both uniform and nonuniform sampling to improve exploration efficiency. The proposed RRT*-NUS method is evaluated against six baseline algorithms: RRT*, Informed RRT*, RRT*-N (normal sampling RRT*), GS-RRT* (goal-oriented sampling RRT*), DR-RRT* (directional random sampling RRT*), and hybrid-RRT* in three different 384*384 2D simulation scenarios. The numerical simulation results indicate that the proposed RRT*-NUS surpasses the baseline RRT* algorithms in terms of planning time and convergence. It outperforms RRT* by 67.5% and Hybrid RRT* by 54% in time performance. Additionally, it achieves a convergence rate of 0.41 units/s, which is 3× faster than RRT* and almost 2× faster than Hybrid RRT*.

## Introduction

Autonomous mobile robots (AMR) are used for a wide range of applications, such as industrial automation^1^, healthcare^2^, social^3^, and agriculture^4^. An AMR can perform these tasks based on its path planning (PP) algorithm, which needs to be effective and efficient so the AMR can reach its destination while avoiding obstacles. Sampling-based algorithms have gained prominence in mobile robot path planning due to their ability to solve complex, high-dimensional problems^5^. Popular algorithms include rapidly-exploring random trees (RRT)^6^, Probabilistic Roadmaps (PRM)^7^, and their enhanced versions of RRT* and PRM*. Because of its higher performance in real-world applications, RRT has sparked greater attention in the robotics community than PRM^8^. It creates a tree starting with the initial and ending with the final configuration. Over the last decade, increased attention has resulted in a proliferation of RRT variants, which are discussed in review papers^9–12^. The RRT*^13^ algorithm is a significant advancement and the most notable version of the RRT^14^. These algorithms offer optimal solutions with probabilistic completeness. Recent RRT* path planning algorithms primarily focus on improving path quality, rapid planning, and robustness in complex dynamic environments. ATS-RRT* enhances initial path planning and path quality through alternative paths and triangular area sampling strategies^15^. R2-RRT* incorporates mobility reliability metrics to address uncertain terrain conditions for off-road autonomous vehicles^16^. Target Tree-RRT* utilizes clothoid paths and a cost function to generate continuous curvature paths for autonomous parking, improving accuracy and success rates in cluttered environments^17^. Agile-RRT* introduces an adaptive goal-biased sampling strategy and a path optimization approach using a secondary tree and subset-informed sampling, significantly reducing initial solution search time and sub-optimal solution search time compared to RRT*^18^. These variants demonstrate improved performance in various scenarios, including narrow passages, cluttered obstacles, and uncertain terrains, addressing key challenges in path planning for autonomous vehicles and mobile robots.The RRT* based PP algorithm uses a uniform sample technique for its sampling process, which is an important component. Within C-space, it connects to the nearby node in the tree if there is open space, otherwise it selects a random sample. Any PP system that uses sampling must be able to quickly converge^19^. A significant disadvantage of RRT* is that, while it creates an optimal path, it does not guarantee fast convergence^20–22^. RRT* is slow to converge since it searches the whole configuration space with uniform sampling^23,24^. The RRT* algorithm’s sampling mechanism is critical for both search exploration and convergence speeds^25^. Non-uniform sampling methods can be utilized to address the slow convergence issue with RRT*^26,27^. When opposed to uniform sampling, non-uniform sampling prefers portions of the search zone and hence more likely to sample them. A detailed assessment of the non-uniform sampling used by the RRT* technique was found in the literature^28^. Slow convergence is an unavoidable consequence of RRT*’s use of uniform sampling to search the complete configuration. To boost the convergence rate, a goal-biased sampling strategy in RRT* is proposed that uses random gradient descent heuristics and artificial potential functions^29,30^. Also, goal-directed technique^31^ employs goal heuristics; it generates two random samples simultaneously and adds one closer to the target point. A goal-oriented PP algorithm uses the node-counting method to generate more samples near the objective region^32^. However, goal-based sampling has various disadvantages, including greater processing complexity and the risk of becoming trapped in a difficult environment. Informed sampling techniques have significantly improved optimal PP algorithms. Gammell et al. introduced Informed RRT*^33^, which directly samples the informed set to overcome the curse of dimensionality in high-dimensional spaces and analyzes minimum-path-length problems using the L2 (Euclidean) norm, and shows that existing approaches become less effective as the state dimension increases, at a rate faster than exponential. Algorithms based on Informed RRT* use hyper-ellipsoids and direct-constrained sampling approaches to extract samples from the configuration space^34^. The Batch Informed Trees (BIT*) algorithm has been improved to take into account energy costs for reconfigurable robots, leading to faster results and more energy-saving paths^35^. Another study proposed the bidirectional Informed-RRT* (BI-RRT*) algorithm, which incorporates an extended range, dual-direction exploration, and trajectory refinement to enhance PP capabilities and safety^36^. However, Informed RRT* based algorithms use the RRT* uniform sampling method to create an initial path throughout the entire configuration space. An approach to adaptive sampling RRT*N^37^ selects samples from the configuration space near the line linking the initial and final configurations with a normal distribution. Furthermore, it cannot generate trails when space is restricted. As a result, when compared to uniform sampling in RRT*, non-uniform sampling increases the convergence rate while decreasing the number of random nodes needed to identify a route^38,39^. On the other side, it causes local minima, constrained exploration, and an increase in computational complexity. Thus, it may be unable to generate a path in complex, obstacle-filled environments.

According to the literature, RRT* produces optimal pathways by uniform sampling; yet, convergence is slow due to exhaustive exploration. Nonuniform sampling, on the other hand, converges faster but, due to its limited exploration, may fail to identify a solution in each environment. As a result, RRT* PP algorithms must select a sampling strategy that is neither excessively exploitative nor overly exploratory^40,41^. To achieve a better balance between excessive exploitation and exploration, mixed non-uniform and uniform sampling strategies in RRT* through hybrid sampling schemes have become a key focus of current research. It has the potential to significantly enhance the performance of RRT* in AMR navigation while considerably shortening the convergence rate. A novel sampling method, called DR-RRT*^42^, is proposed that combines directional non-uniform sampling with uniform sampling. Combining directional sampling with uniform sampling reduces the search space and improves path optimality. Another method, named GS-RRT*^43^, combines goal-oriented non-uniform sampling with uniform sampling to reduce unnecessary sample exploration by collecting more samples near the goal, which helps improve success rates and convergence. Further, Hybrid-RRT*^44^ is proposed that utilize a hybrid sampling method that combines non-uniform normal sampling with uniform sampling. It effectively solves the problem of slow progress in RRT* that comes from using uniform sampling and the lack of exploration seen in non-uniform sampling methods. Even though hybrid sampling methods that mix non-uniform and uniform strategies have made progress in finding better paths and using nodes more efficiently, there is still a research gap to improve how quickly they reach a solution with further less node exploration and improve the convergence in RRT*. Current methods still exhibit scope for optimization in terms of reducing computational overhead and achieving faster convergence.

This study presents a novel path planning algorithm called RRT*-NUS, which combines three different non-uniform sampling methods—directed, normal, and goal-oriented—with uniform sampling. By employing a hybrid sampling framework, RRT*-NUS is designed to adapt effectively to complex environments while enhancing convergence speed. This approach addresses the limitations of conventional sampling techniques and offers significant potential for improving path-planning performance in AMRs. The proposed research work’s major contributions are mentioned below:

- A hybrid sampling technique combines the benefits of uniform and non-uniform sampling.
- A non-uniform sampling scheme combines normal, directional, and goal-oriented samplers.
- A goal-directed selection strategy to balance uniform and non-uniform samplers.

This research article has the following structure: “Problem definition” section depicts the definition of the PP problem. In “Related work” section shows the basic RRT* algorithms used for comparison, and “Proposed RRT*-NUS framework” section explains the methodology of the proposed RRT*-NUS algorithm. “Results and discussion” section will discuss the results and analysis of the RRT*-NUS PP approach. The article concludes in “Conclusions and future work” section by presenting the research outcomes and potential future possibilities for the field.

## Problem definition

Consider a configuration $x \in \mathscr{C}$ that depicts the location of the robot. The space with obstacles is expressed by $\mathscr{C}_{\text{obs}}$, and the space without obstacles is specified by $\mathscr{C}_{\text{free}} = \mathscr{C} {\setminus} \mathscr{C}_{\text{obs}}$.

Let $x_{\text{start}} \in \mathscr{C}_{\text{free}}$ be the start node and $x_{\text{goal}} \in \mathscr{C}_{\text{free}}$ be the goal (target) node. The path over configuration space is defined as:

$$T: [0,1] \rightarrow \mathscr{C}$$

If $T(\tau ) \in \mathscr{C}_{\text{free}} \; \forall \tau \in [0,1]$, then the path $T$ is said to be *collision-free*.

The path planning (PP) problem aims to find a collision-free path:

$$T: [0,1] \rightarrow \mathscr{C}_{\text{free}}$$

such that $T(0) = x_{\text{start}}$, $T(1) \in x_{\text{goal}}$, and $T(\tau ) \in \mathscr{C}_{\text{free}} \; \forall \tau \in [0,1]$. A path is considered *realistic* if:

$$T: [0,1] \rightarrow \mathscr{C}, \quad T(0) = x_{\text{start}}, \quad T(1) \in x_{\text{goal}}, \quad \text{and} \quad T(\tau ) \in \mathscr{C}_{\text{free}}.$$

*Challenge 1 (Feasible PP)* For a given PP problem $(x_{\text{start}}, x_{\text{goal}}, \mathscr{C}_{\text{free}})$, find a realistic (collision-free) path, if it exists. If such a path does not exist, declare failure.

Let $\mathscr{P}$ denote the set of all paths over $\mathscr{C}$, and let $\mathscr{P}_{\text{feasible}} \subset \mathscr{P}$ denote the set of all realistic (feasible) paths.

*Challenge 2 (Optimal PP)* For a given PP problem $(x_{\text{start}}, x_{\text{goal}}, \mathscr{C}_{\text{free}})$, and a cost function $\text{cst}: \mathscr{P} \rightarrow \mathbb {R}_{\ge 0}$, find an optimal realistic path $T^* \in \mathscr{P}_{\text{feasible}}$ such that:

$$\text{cst}(T^*) = \min \{ \text{cst}(T) \mid T \in \mathscr{P}_{\text{feasible}} \}$$

If such a path does not exist, declare failure.

## Related work

This section presents the baseline algorithms considered for comparative analysis, as detailed in “Results and discussion” section. The baseline algorithms consist of uniform sampling methods like standard RRT*^13^, non-uniform sampling methods such as RRT*-N^37^ and informed RRT*^33^, and a mix of methods called hybrid sampling, which includes DR-RRT*^42^, GS-RRT*^43^, and Hybrid-RRT*^44^.

### RRT* algorithm

An extension of the basic RRT algorithm for PP in high-dimensional spaces is the RRT* (rapidly exploring random tree star) algorithm. RRT is designed to find a feasible path, while RRT* focuses on finding the optimal path in terms of the shortest distance or minimum cost. Algorithm 1 describes the RRT* algorithm, which generates random samples using a uniform distribution. The basic procedure used in the RRT*^13^ is described below.

*Sampling* A uniform random sample $x_{\text{rand}}$ is generated from the configuration space (C-space) excluding obstacles using the sample() function (Step 3).

*Distance* The cost of the path that connects two nodes is returned under the condition that the region between the nodes is devoid of any obstacles. Euclidean distance (*d*) is used to express the distance.

*Nearest neighbor* The nearest() function is responsible for locating the node $x_{\text{nearest}}$ from the list of vertices, *v* is closest to $x_{\text{rand}}$ by utilizing the Euclidean distance (Step 4).

*Steer* The steer() function is accountable for driving the system along the path that provides $x_{\text{new}}$ at a $\delta$ from $x_{\text{nearest}}$ to reach $x_{\text{rand}}$ (Step 5).

*Collision detection* The ObstacleFree() function is accountable for determining whether or not the path *T* is located in the zone that is free of obstacles (Step 6).

*Nearby vertices* If the vertex $x_{\text{new}}$ and the edge joining it to $x_{\text{nearest}}$ are in the obstacle-free zone, then the near_nodes_() function will return the nodes that are nearby and within a ball with a volume of $\beta (\log n)/n$ around $x_{\text{near}}$, where $\beta$ is constant (Step 7).

*Choose parent* The updateParent() function selects the parent vertex of a new vertex $x_{\text{new}}$ from the nearby vertices (Step 8).

*Insert node* The vertex and edge are added to the tree *g* with the add() function (Step 9).

*Rewire* rewire() function determines whether the cost to nodes in $x_{\text{near}}$ is lower via $x_{\text{new}}$ than previous costs. If lower-cost, then $x_{\text{parent}}$ is changed to $x_{\text{new}}$ (Step 10).

*Update* When creating a new vertex, the updateParent() function selects a parent vertex from the surrounding vertices. The add() function is then used to add the vertex and edge to tree *g*. Hence, the rewire() function is used to examine the cost to the vertex and find the path in the tree, *g* from $x_{\text{start}}$ to $x_{\text{goal}}$.

Steps 5 to 12 repeat until the intended goal, or maximum sample size (K), is reached.

**Algorithm 1:**
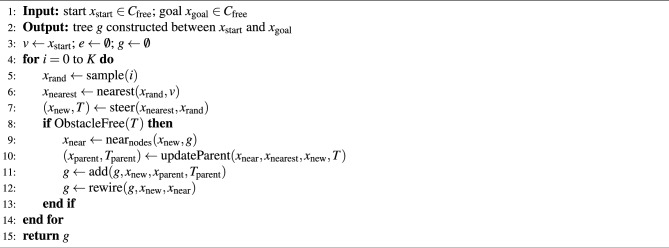
RRT* framework

### Informed RRT* algorithm

Algorithm 2 outlines the Informed-RRT* process. The key distinction from standard RRT* lies in the informedsample() function. This method uses a smart sampling technique with a hyperellipsoid that targets areas closer to the goal. By restricting the sampling area to this hyperellipsoid, Informed-RRT* reaches the best solution more quickly with fewer samples.

**Algorithm 2:**
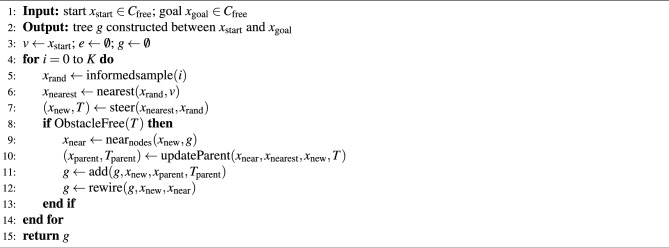
Informed-RRT^*^ framework

### RRT*-N algorithm

Algorithm 3 describes the RRT*-N algorithm, which employs a normal distribution to generate random samples. The RRT*-N algorithm specifically designs the normalsample() function to guide sampling more effectively, unlike the sampling functions used in RRT* and Informed-RRT*. It constructs an imaginary line between the start and goal configurations and samples points from a normal distribution centered along this line, avoiding obstacle regions. This bias toward the mean helps steer the sampling process closer to the optimal path, enhancing exploration in promising regions while maintaining some degree of global search. As a result, the algorithm achieves faster convergence toward an optimal solution.

**Algorithm 3:**
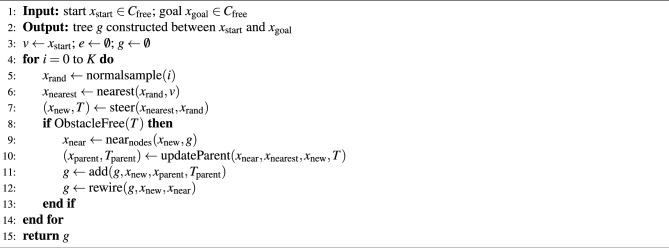
RRT^*^-N framework

### DR-RRT* algorithm

The directional random sampling RRT* (DR-RRT*) algorithm is an enhanced variant of the RRT* method, tailored for AMR path planning with a focus on direction and orientation. Its primary goal is to improve planning performance by embedding directional constraints into the sampling strategy. Algorithm 4 outlines the structure of the DR-RRT* framework^42^. The tree expands incrementally from the start to the goal configuration using a combination of non-uniform adaptive sampling and uniform sampling procedures. The directional sampling method reduces the sampling space by forming an elliptical heuristic among the start and goal configurations.

**Algorithm 4:**
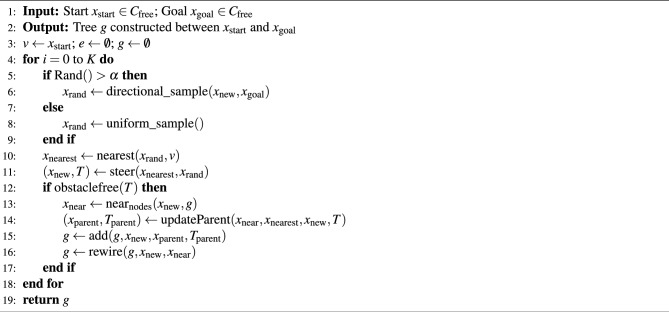
DR-RRT* framework

### GS-RRT* algorithm

The goal-oriented sampling RRT*(GS-RRT*) algorithm is a modified version of RRT* designed to enhance path planning performance and convergence speed by prioritizing sampling in regions closer to the goal. This approach is especially beneficial for AMRs, where efficient navigation toward a goal is essential. The structure of the GS-RRT* framework is outlined in Algorithm 5^43^.

**Algorithm 5:**
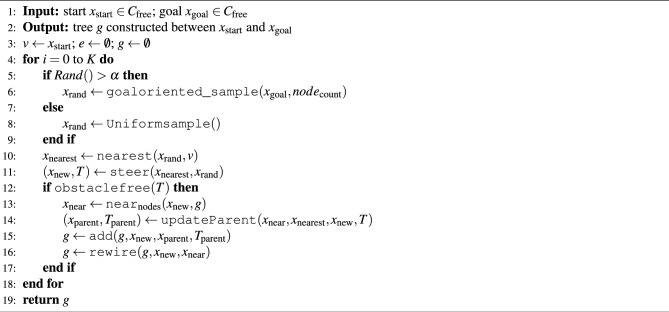
GS-RRT* framework

### Hybrid-RRT* algorithm

The Hybrid-RRT* framework integrates both uniform and non-uniform sampling to accelerate convergence^44^. The Hybrid-RRT* Algorithm is presented in Algorithm 6. The Hybrid-RRT* algorithm enhances the traditional RRT* by incorporating a hybrid sampling strategy to combine both uniform and non-uniform (Gaussian-based) sampling approaches. In each iteration, a random number is generated, and if this number exceeds threshold $\alpha$, the algorithm employs a hybrid strategy where one sample is drawn from a normal distribution and another from a uniform distribution. These samples are then evaluated based on their proximity to the goal, and the closest sample is selected for expansion using a goal-oriented heuristic. Conversely, if the random number is less than threshold $\alpha$, the algorithm defaults to uniform sampling. This adaptive sampling mechanism increases the likelihood of sampling nodes that lie closer to the goal region, thereby enhancing the search’s efficiency. By combining focused sampling (non-uniform sampling) and broad sampling (uniform sampling), Hybrid-RRT* produces higher quality paths and reaches solutions more quickly in complicated and multi-dimensional spaces.

**Algorithm 6:**
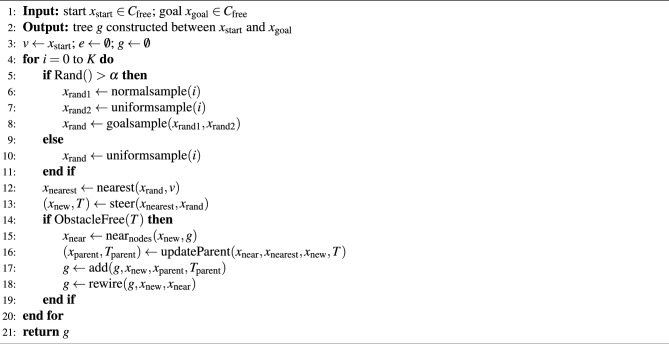
Hybrid-RRT* framework

**Fig. 1:**
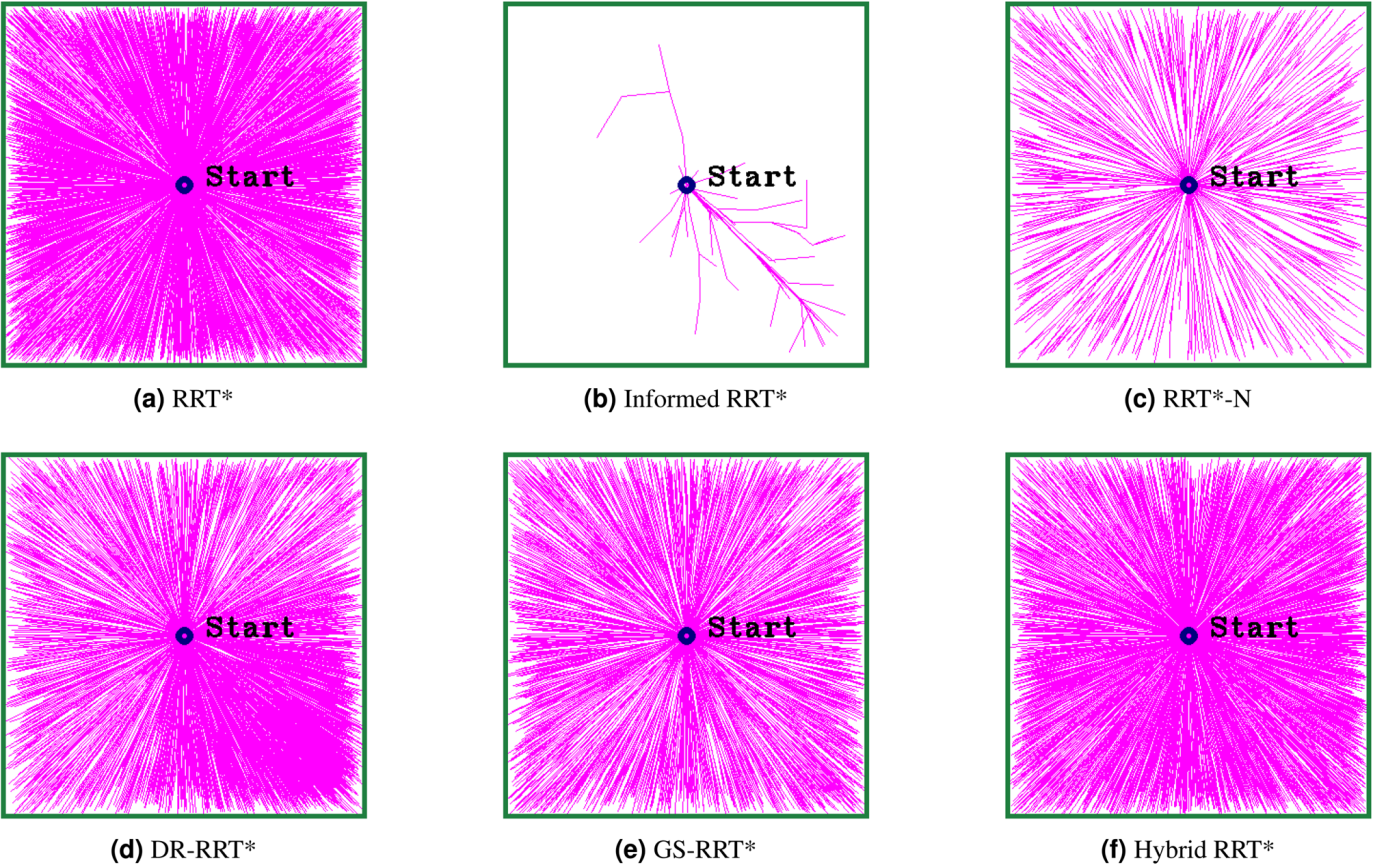
Tree exploration patterns after 10,000 iterations for various RRT* baseline algorithms.

Figure 1 shows the exploration of the various RRT* baseline algorithms after 10,000 iterations, highlighting differences in their sampling strategies. The classic RRT* exhibits a uniform exploration without directional preference from the start point, covering the space extensively. In contrast, the Informed RRT* algorithm concentrates its exploration in an elliptical area between the start and goal points, showing its exploration. RRT*-N exhibits slightly better-directed growth compared to RRT*, while still maintaining broad exploration. DR-RRT* and GS-RRT* create trees that are closer together and more organized, showing that they adapt their sampling based on the situation. Hybrid RRT* produces a well-balanced tree that efficiently covers space while maintaining directionality.

## Proposed RRT*-NUS framework

**Fig. 2:**
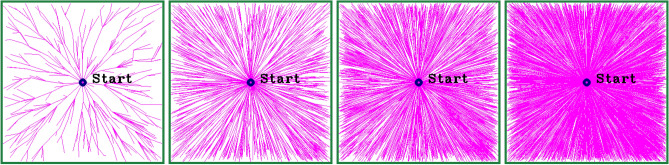
Tree exploration by RRT*-NUS (**a**) 1000 (**b**) 5000 (**c**) 10,000 (**d**) 20,000.

The Hybrid-RRT* algorithm enhances the exploration process more effectively than the standard uniform RRT*. However, it still doesn’t control the direction of sample exploration, and it only samples at one level each time. These limitations reduce its effectiveness in guiding the search efficiently toward the goal. To solve this problem, the proposed RRT*-NUS framework uses a unique sampling approach that mixes normal, goal-oriented, and direction-based nonuniform samples with uniform sampling methods to help reach the goal more quickly. The proposed RRT*-NUS framework employs a goal-directed strategy to select find random samples from nonuniform sampling methods. The selection of uniform or non-uniform samplers is an important decision in hybrid sampling. The parameter $\alpha$ determines the trade-off between exploration and exploitation in the mixed sampling strategy. The mixed sampling strategy is expressed as:

1 $$\begin{aligned} p(x) = \alpha \cdot p_{\text{random}}(x) + (1 - \alpha ) \cdot p_{\text{biased}}(x) \end{aligned}$$

where *p*(*x*)—overall sampling distribution $p_{\text{random}}(x)$—uniform random sampling probability $p_{\text{biased}}(x)$—Biased sampling probability guided by a heuristic function. $\alpha$—weighing factor that controls the balance between exploration and exploitation. strategy. An $\alpha$ value of 0.5 is selected, following the recommendation in^45^, to provide equal emphasis on exploration and exploitation. Simulations also indicate that $\alpha = 0.5$ offers a balanced trade-off between the two competing objectives. Algorithm 7 describes the pseudocode for the RRT*-NUS. Three different non-uniform sampling methods are combined with the uniform sampling method using a simple arbitrary selection method Rand() > *α*. If the condition Rand() > *α* (Step 3) is satisfied, three samples $x_{\text{rand1}}, x_{\text{rand2}}$, and $x_{\text{rand3}}$ are generated using the *directional sample* procedure (Step 4), *normal sample* procedure (Step 5), and *goal-oriented sample* procedure (Step 6), respectively. A node that is the near node to the goal is selected using the near_*i*_ function (Step 7). If the condition fails, $x_{\text{rand}}$ is generated using the *uniform sample* procedure (Step 9). The node is attached to the tree, which grows step by step until it reaches the target or the maximum sample size (*K*) is reached. Figure 2 illustrates the progressive tree expansion of the RRT*-NUS algorithm at four different iteration: 1000, 5000, 10,000, and 20,000. At 1000 iterations, the tree appears relatively sparse with limited branches extending from the start point. By 5000 iterations, the coverage improves significantly, showing denser branching in all directions. As the iteration count increases to 10,000, the tree structure becomes more uniformly distributed, indicating efficient space exploration. At 20,000 iterations, the exploration reaches near-complete coverage, with the tree densely populated and well-expanded across the entire area. This demonstrates the algorithm’s ability to explore the configuration space more effectively with increasing iterations.

**Algorithm 7:**
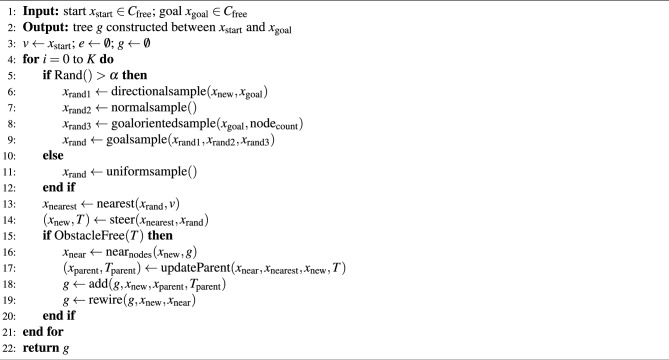
RRT*-NUS framework

### Probabilistic extensiveness of RRT*-NUS

#### Theorem 1

(RRT*-NUS Completeness) The RRT*-NUS algorithm is Probabilistically comprehensive. For a given PP problem $(x_{\text{start}}, x_{\text{goal}}, C_{\text{free}})$ t*here exist constants* $w > 0$ *and* $t_0 \in \mathbb {R}$ *such that*

2 $$\begin{aligned} \mathbb {P}\left( V^{\text{RRT}^*\text{-NUS}} \cap x_{\text{goal}} \ne \emptyset \right)> 1 - e^{-w t}, \quad \forall t > t_0 \end{aligned}$$

*where* $V^{\text{RRT}^*\text{-NUS}}$ is the set of nodes generated by the RRT*-NUS algorithm.

#### Proof

The RRT*-NUS proceeds an associated graph for a given environment, so the result supports the probabilistic extensiveness of RRT* $\square$

### Analysis of complexity

The space and time complexity of the RRT*-NUS is explained through the big-$\mathscr{O}$ notation. It postulates the amount of space and time expected. Certain functions *f*(*t*) and *h*(*t*), *f*(*t*) is said to belong to $\mathscr{O}(h(t))$ if

3 $$\begin{aligned} \lim _{t \rightarrow \infty } \left| \frac{f(t)}{h(t)} \right| < \infty \end{aligned}$$

#### Space complexity

Space complexity indicates the measure of memory needed. RRT*-NUS builds a tree $g_n = (v_n, e_n)$, and the size of memory involved by RRT*-NUS is defined by the tree $g_n$.

##### Lemma 1

$$|g_n| \in \mathscr{O}(n)$$.

##### Proof

For RRT*-NUS, tree $|g_n|$ size is computed as $|g_n| = |v_n| + |e_n|$. If the *n* nodes are visited by $g_n$, then $|v_n| = n$, and $|e_n| = n - 1$. Therefore, the size of the tree is

4 $$\begin{aligned} |g_n| = |v_n| + |e_n| = 2n - 1 \end{aligned}$$

$$\square$$

#### Time complexity

The time complexity is calculated by counting the number of procedure calls to the method with the highest time complexity. Take the number of calls to the obstaclefree method by the algorithm be symbolized by $M^{\text{ALG}}_n$ in *n* iterations.

##### Lemma 2

$$M^{\text{RRT*-NUS}}_n \in \mathscr{O}(\log n)$$.

##### Proof

Let $C^{\text{RRT*-NUS}}_n$ and $R^{\text{RRT*-NUS}}_n$ be the number of procedure calls to the obstaclefree procedure in updateParent and rewire methods. Then

5 $$\begin{aligned} M^{\text{RRT*-NUS}}_n = C^{\text{RRT*-NUS}}_n + R^{\text{RRT*-NUS}}_n \end{aligned}$$

Since RRT*-NUS does not amend the updateParent and rewire methods, the complexity of the time is unaltered as RRT^*^, $C^{\text{RRT*-NUS}}_n \in \mathscr{O}(\log n)$ and $R^{\text{RRT*-NUS}}_n \in \mathscr{O}(\log n)$. Hence

$$M^{\text{RRT*-NUS}}_n \in \mathscr{O}(\log n).$$

$$\square$$

### Performance metrics

- *Path length (p)* Represents the length of the path found by the algorithm. Shorter paths are preferable as they indicate efficiency.
- *Time (t)* The time taken by the algorithm to find a feasible solution. Lower time indicates higher computational efficiency.
- *Number of nodes (n)* The number of nodes generated during the planning process. Fewer nodes suggest more efficient sampling.
- *Success rate (SR)* The percentage of trials where a feasible solution was found. Higher success rates indicate reliability.
- *Convergence rate (CR)* Defined as the rate of improvement in path cost per second. Higher values reflect faster convergence toward optimal solutions.

## Results and discussion

**Fig. 3:**
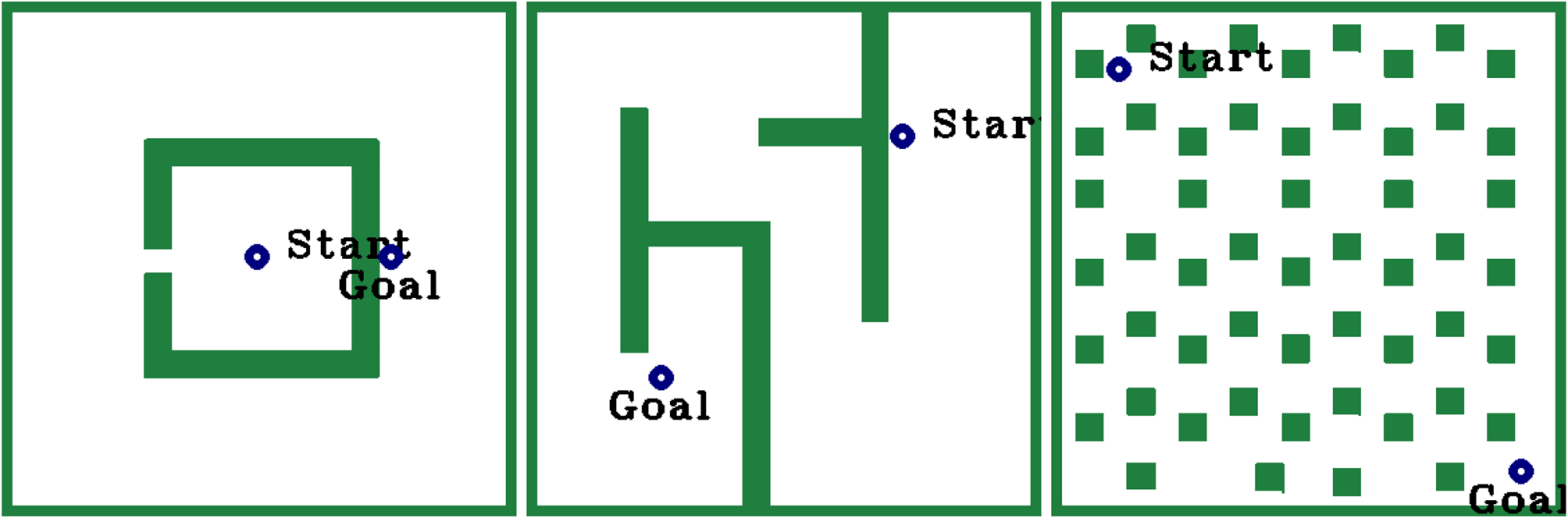
Simulation environments used for evaluating the algorithms: (**a**) Narrow, (**b**) Maze, and (**c**) Cluttered.

To compare the proposed RRT*-NUS to the baseline methods RRT*, Informed RRT*, RRT*-N, DR-RRT*, GS-RRT* and Hybrid-RRT* in three environments are used, each having a 2D map of 384*384 pixels (Fig. 3). These maps are frequently used by researchers to compare various path-planning algorithms.

The proposed method is implemented in C++ and executed on an Intel i5-12500 CPU with 16 GB RAM, running ROS2 Humble on Ubuntu 22.04. The statistical evaluation of each algorithm is based on 100 independent trials, with sampling-based planners relying on fully random sampling as described in prior works^13,32^. Performance is evaluated using five key metrics: path computation time (t), path length (p), number of nodes explored (n), convergence rate (CR), and success rate (SR). These metrics are standard in the literature for benchmarking and comparing path-planning algorithms.

The parameter convergence rate is computed as

6 $$\begin{aligned} \frac{\text{cst}(T_{\text{init}}) - \text{cst}(T_{opt})}{t_{\text{opt}} - t_{\text{init}}} \quad \text{(units/s)} \end{aligned}$$

whereas the initial realistic path computed in $t_{\text{init}}$ is denoted as $T_{\text{init}}$ and the optimal path calculated in $t_{\text{opt}}$ time is $T_{opt}$. The success rate is calculated based on the algorithm’s path creation capability within the maximum samples. It will be considered a failure if it doesn’t generate an optimal path within the maximum sample size (*K*).

**Fig. 4:**
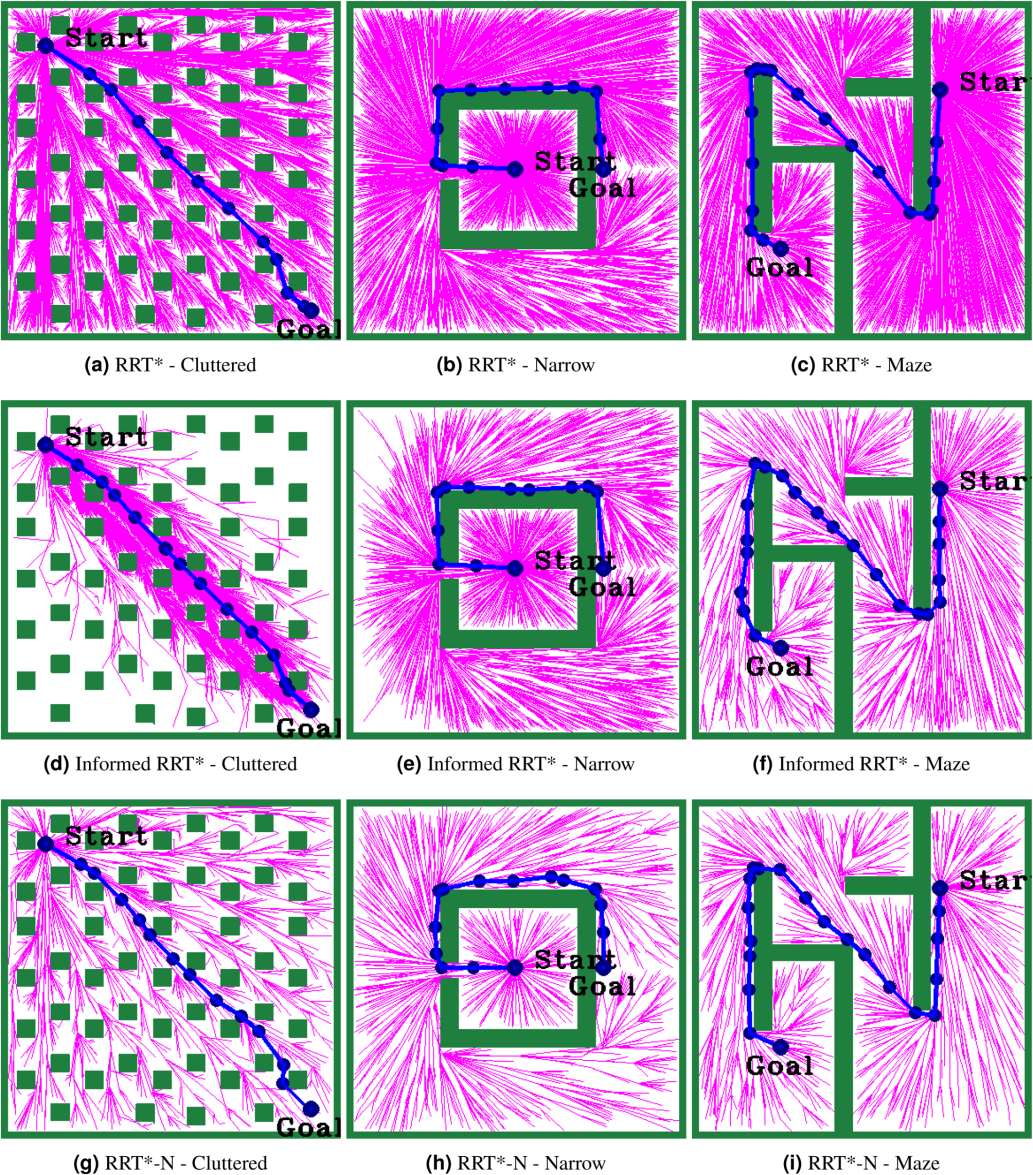
Paths generated by RRT*, Informed RRT*, and RRT*-N across three environments.

**Fig. 5:**
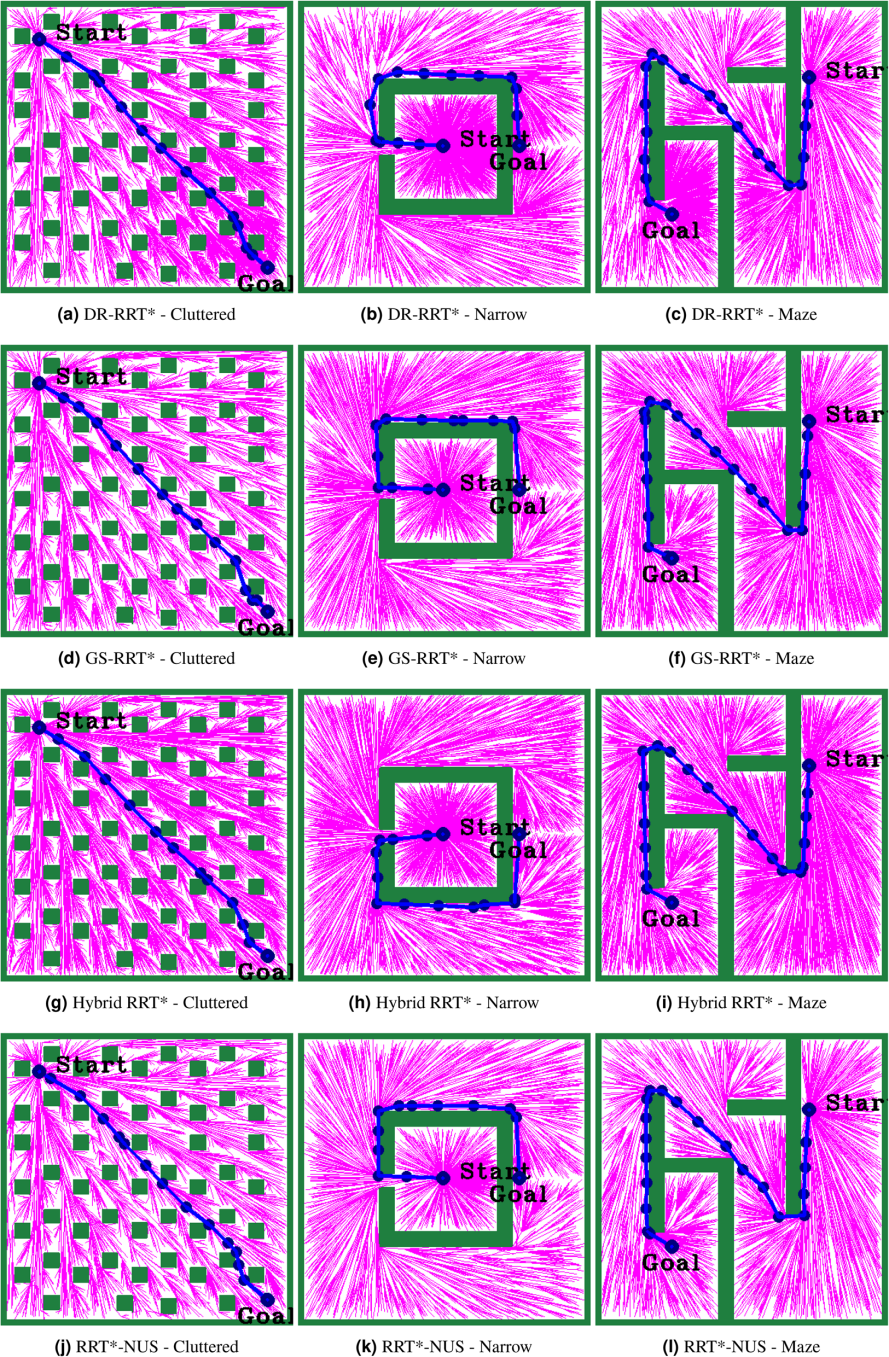
Paths generated by DR-RRT*, GS-RRT*, Hybrid-RRT*, and RRT*-NUS across three environments.

The following different parameters are used to evaluate the proposed research works: Selecting the proper step size in the RRT*-based PP algorithm is important. Considering the contrast of the best performance of the given environmental maps, step size 50 was selected in this proposed work. Another important parameter is the experimental stopping criteria, i.e., the maximum sample size (*K*). The maximum sample size of 10,000 samples was set according to the literature^13,32,37^ for studying the convergence and success rate. The selection of uniform or non-uniform samplers is an important decision in hybrid sampling. A balance value of $\alpha = 0.5$ was chosen based on the simulation study and literature^45^. Figure 4 shows the paths generated by the baseline algorithms RRT*, Informed RRT*, and RRT*-N across Cluttered, Narrow, and Maze environments. RRT* explores widely, resulting in longer paths. Informed RRT* improves goal-directed search, producing shorter paths. RRT*-N consistently shows the most efficient and focused exploration, yielding smoother and more optimal paths in all scenarios. Figure 5 shows the results of different hybrid sampling path planning methods like DR-RRT*, GS-RRT*, Hybrid RRT*, and RRT*-NUS in crowded, tight, and maze-like areas. In cluttered environments, all algorithms demonstrate enhanced goal-directed growth, with GS-RRT* and Hybrid RRT* offering balanced exploration and path smoothness. In narrow passages and maze environments, RRT*-NUS exhibits the most efficient and direct path generation, effectively navigating complex structures with minimal deviation. Overall, RRT*-NUS and Hybrid RRT* outperform others in maintaining optimal paths while handling diverse obstacle distributions.

### Comparative performance analysis of RRT variants

**Fig. 6:**
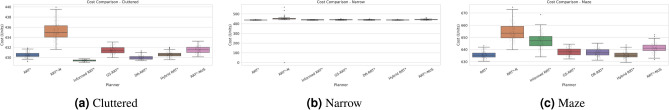
Path cost comparison across different environments using various RRT* planner variants.

**Fig. 7:**
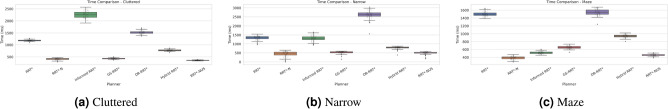
Time comparison across different environments using various RRT* planner variants.

**Fig. 8:**
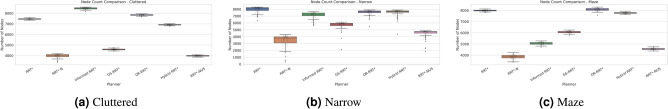
Node count comparison across different environments using various RRT* planner variants.

**Fig. 9:**
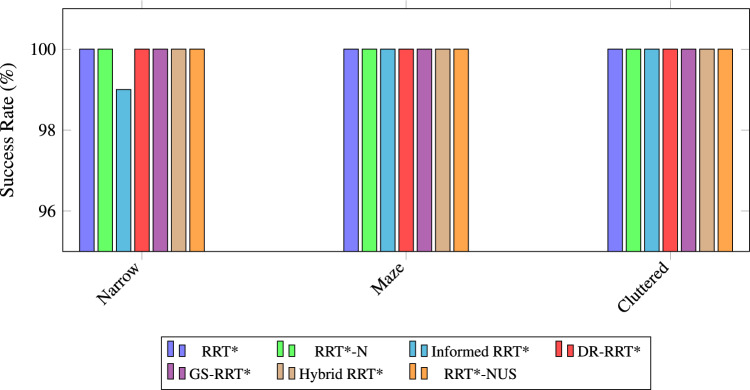
Success rate across different environments using various RRT* planner variants.

**Fig. 10:**
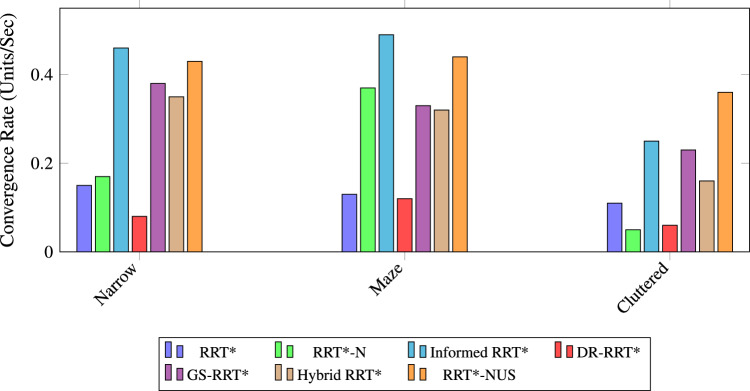
Convergence rate across different environments using various RRT* planner variants.

The performance comparison in Table 1 comprehensively evaluates several widely used sampling-based path planning algorithms across three representative environments: narrow passages, maze-like structures, and cluttered. The metrics considered include success rate, convergence rate, average path cost, average planning time, and average number of nodes visited. Figure 6 shows the path planning cost performance of various RRT* variants across Cluttered, Narrow, and Maze environments. Overall, the path cost analysis reveals that Hybrid RRT* and Informed RRT* offer the most consistent and cost-efficient performance across all environments. In terms of path optimality, the proposed RRT*-NUS performs competitively with other algorithms. Path Planning time serves as a critical metric for path planning. This substantial reduction highlights their suitability for time-critical applications, where rapid decision-making is essential. Figure 7 shows time comparisons for different RRT* planner variants across three types of environments. The analysis reveals that RRT-NUS* consistently outperforms all other planners. It demonstrates significantly lower path planning times, achieving up to 67.5% compared to RRT* and 54% reduction compared to Hybrid RRT*. The average number of nodes visited offers valuable insight into the computational efficiency of each path-planning algorithm. Figure 8 illustrates the node count comparisons for various RRT* planner variants across three different environments. RRT-N* and RRT-NUS* consistently demonstrate reduced node usage across all environments, indicating better planning efficiency and goal-directed behavior. RRT*-NUS shows a significant advantage, visiting only 4344 nodes on average, which is nearly 55% fewer than standard RRT*. The bar plot in Fig. 9 the Success Rate performance of different RRT* variants—RRT*, RRT*-N, Informed RRT*, DR-RRT*, GS-RRT*, Hybrid RRT*, and RRT*-NUS—across Narrow, Maze, and Cluttered environments. All algorithms exhibit a strong success rate of 100%, except RRT*-N in the narrow scenario, where it achieves a slightly lower success rate of 99%. Though RRT*-N path cost performance is superior to the proposed RRT*-NUS, it fails to achieve success in the narrow environment, which is the major limitation of this algorithm. The bar chart in Fig. 10 illustrates the Convergence Rate performance of various RRT* state-of-the-art planner variants with the proposed RRT*-NUS across three environments. The major issue with RRT*, which is the convergence rate, is effectively addressed by the proposed RRT*-NUS, which reaches an average CR of 0.41 units/s—over three times faster than RRT* and almost double that of Hybrid RRT*. Among all evaluated planners, the proposed RRT-NUS* stands out as particularly effective and well-balanced. It consistently delivers high success rates, competitive path quality, low planning time, and minimal node generation. This makes it highly suitable for applications requiring fast and efficient planning, such as mobile robot navigation, real-time obstacle avoidance, and dynamic task execution in complex environments.

**Table 1:** Performance comparison of path planning algorithms across different environments.

Algorithm	Environment	Success rate (%)	Avg. cost	Avg. time (ms)	Avg. nodes visited	CR (units/s)
RRT*^13^	Narrow	100	437.46	1331.59	7954.12	0.15
	Maze	100	635.58	1499.04	7987.53	0.13
	Cluttered	100	430.51	1187.96	7461.63	0.11
	Average performance	100	501.18	1339.53	7801.09	0.13
Informed RRT*^33^	Narrow	100	439.25	1310.08	7136.61	0.17
	Maze	100	647.59	516.69	5056.67	0.37
	Cluttered	100	429.42	2253.17	8454.91	0.05
	Average performance	100	505.42	1360.65	6882.73	0.20
RRT*-N^37^	Narrow	99	448.89	440.72	3440.37	0.46
	Maze	100	654.53	386.27	3846.78	0.49
	Cluttered	100	435.22	412.67	3993.5	0.25
	Average performance	99.67	512.88	413.22	3759.55	0.40
DR-RRT*^42^	Narrow	100	439.01	2628.6	7559.39	0.08
	Maze	100	637.52	1545.16	8078.75	0.12
	Cluttered	100	429.96	1522.99	7837.4	0.06
	Average performance	100	502.16	1898.92	7825.18	0.09
GS-RRT*^43^	Narrow	100	439.72	523.78	5698.52	0.38
	Maze	100	638.1	650.38	6064.94	0.33
	Cluttered	100	431.47	434.2	4583.75	0.23
	Average performance	100	503.10	536.12	5449.07	0.31
Hybrid RRT*^44^	Narrow	100	437.84	785.96	7538.69	0.35
	Maze	100	635.55	941.99	7768.07	0.32
	cluttered	100	430.6	771.81	6910.4	0.16
	Average performance	100	501.33	833.25	7405.72	0.27
RRT*-NUS (proposed)	Narrow	100	441.6	491.72	4497.62	0.43
	Maze	100	641.23	457.47	4553.88	0.44
	Cluttered	100	431.52	357.15	3981.44	0.36
	Average performance	100	504.78	435.45	4344.31	0.41

The comparison of statistical test results in Table 2 shows that the RRT*-NUS algorithm works better than baseline RRT* path planning methods. The proposed RRT*-NUS demonstrates a consistently significant improvement in planning time performance, with *p* values far below the 0.05 threshold, confirming faster planning compared to RRT*, Informed RRT*, RRT*-N, DR-RRT*, GS-RRT*, and Hybrid RRT*. In terms of node exploration, RRT*-NUS shows a notable reduction in the number of nodes visited, particularly in the cluttered and maze environments. The convergence rate (CR) also shows very significant differences in all environments, which proves that RRT*-NUS is a planner that converges faster than baseline algorithms. While path cost differences are statistically insignificant, this suggests that RRT*-NUS maintains path quality on par with existing methods. Overall, the analysis validates that RRT*-NUS achieves superior planning efficiency without compromising path optimality.

**Table 2:** Statistical comparison of RRT*-NUS against baseline algorithms across environments.

Compared with	Environment	Parameter	*p* value	Reject ($p < 0.05$)
RRT*	Narrow	Cost	0.3311	No
		Time	0.0019	Yes
		Nodes	$7.65 \times 10^{-111}$	Yes
		CR	$4.51 \times 10^{-17}$	Yes
	Maze	Cost	0.1263	No
		Time	0.00087	Yes
		Nodes	$8.15 \times 10^{-277}$	Yes
		CR	$4.15 \times 10^{-26}$	Yes
	Cluttered	Cost	0.5219	No
		Time	0.00013	Yes
		Nodes	0	Yes
		CR	$5.34 \times 10^{-24}$	Yes
Informed RRT*	Narrow	Cost	–	No
		Time	$1.86 \times 10^{-4}$	Yes
		Nodes	$6.61 \times 10^{-7}$	Yes
		CR	$7.53 \times 10^{-91}$	Yes
	Maze	Cost	–	No
		Time	$1.59 \times 10^{-25}$	Yes
		Nodes	$1.50 \times 10^{-16}$	Yes
		CR	$6.58 \times 10^{-82}$	Yes
	Cluttered	Cost	–	No
		Time	$4.28 \times 10^{-11}$	Yes
		Nodes	$1.44 \times 10^{-54}$	Yes
		CR	$1.95 \times 10^{-267}$	Yes
RRT*-N	Narrow	Cost	0.3197	No
		Time	0.5151	No
		Nodes	0.1338	No
		CR	$1.71 \times 10^{-20}$	Yes
	Maze	Cost	–	No
		Time	0.0709	No
		Nodes	$2.67 \times 10^{-36}$	Yes
		CR	$1.93 \times 10^{-69}$	Yes
	Cluttered	Cost	–	No
		Time	$7.65 \times 10^{-4}$	Yes
		Nodes	$1.16 \times 10^{-39}$	Yes
		CR	0.4521	No
DR-RRT*	Narrow	Cost	–	No
		Time	0.0258	Yes
		Nodes	$4.79 \times 10^{-8}$	Yes
		CR	$3.03 \times 10^{-101}$	Yes
	Maze	Cost	–	No
		Time	$2.86 \times 10^{-6}$	Yes
		Nodes	$2.98 \times 10^{-13}$	Yes
		CR	$6.90 \times 10^{-210}$	Yes
	Cluttered	Cost	–	No
		Time	$2.57 \times 10^{-5}$	Yes
		Nodes	$1.39 \times 10^{-44}$	Yes
		CR	$3.57 \times 10^{-307}$	Yes
GS-RRT*	Narrow	Cost	–	No
		Time	0.0246	Yes
		Nodes	$1.11 \times 10^{-4}$	Yes
		CR	$2.53 \times 10^{-39}$	Yes
	Maze	Cost	–	No
		Time	$7.52 \times 10^{-5}$	Yes
		Nodes	$6.69 \times 10^{-10}$	Yes
		CR	$1.32 \times 10^{-185}$	Yes
	Cluttered	Cost	–	No
		Time	$4.32 \times 10^{-5}$	Yes
		Nodes	0.5696	No
		CR	$4.64 \times 10^{-149}$	Yes
Hybrid RRT*	Narrow	Cost	–	No
		Time	0.0032	Yes
		Nodes	$4.76 \times 10^{-15}$	Yes
		CR	$8.65 \times 10^{-102}$	Yes
	Maze	Cost	–	No
		Time	$4.03 \times 10^{-7}$	Yes
		Nodes	$1.23 \times 10^{-26}$	Yes
		CR	$8.83 \times 10^{-274}$	Yes
	Cluttered	Cost	–	No
		Time	$6.05 \times 10^{-6}$	Yes
		Nodes	$1.36 \times 10^{-21}$	Yes
		CR	$3.34 \times 10^{-297}$	Yes

## Conclusions and future work

This study introduces a novel hybrid sampling strategy that effectively addresses the trade-off between exploration and exploitation by combining uniform and non-uniform sampling techniques. The proposed RRT*-NUS algorithm maintains both asymptotic optimality and probabilistic completeness, with computational complexity equivalent to that of standard RRT*. The performance of RRT*-NUS is compared to several other algorithms—specifically, RRT*, RRT*-N, Informed RRT*, DR-RRT*, GS-RRT*, and Hybrid RRT*—in three different environments. Experimental results indicate that RRT*-NUS delivers competitive performance in terms of path optimality while significantly reducing planning time. It consistently records the lowest computation times, with reductions of up to 67.5% compared to RRT* and 54% compared to Hybrid RRT*. Furthermore, it generates fewer nodes than the baseline methods. RRT*-NUS achieves a 100% success rate across all tested environments, equaling or exceeding the performance of other planners (e.g., RRT*-N achieves 99% in narrow passages). One of the key limitations of RRT*—its slow convergence rate—is substantially mitigated by RRT*-NUS, which demonstrates an average convergence rate of 0.41 units/s, over three times faster than RRT* and nearly twice as rapid as Hybrid RRT*. Overall, the RRT*-NUS planner strikes an excellent balance between efficiency, reliability, and path quality. It surpasses state-of-the-art approaches in terms of speed and convergence while maintaining strong performance in path length and success rate. Its adaptability to varied environments and efficient use of computational resources make it a strong candidate for real-world motion planning applications. The proposed algorithm is well-suited for real-time deployment as a global path planner, integrated with a local planner to manage dynamic obstacles. The approach can be effectively implemented within the Robot Operating System 2 (ROS2) framework, utilizing 2D LiDAR for obstacle detection and Simultaneous Localization and Mapping (SLAM) for environment mapping. Future work can explore using the proposed method as a local planner. It can also be extended to 3D environments to support tasks like autonomous navigation, rescue missions, and precise robotic actions. In addition, improvements such as path refinement and handling real-time changes in obstacles and goals can be considered.

## Data Availability

The datasets generated during and/or analyzed during the current study are available from the corresponding author upon reasonable request.
